# Enhanced Muscle Fibers of *Epinephelus coioides* by *Myostatin* Autologous Nucleic Acid Vaccine

**DOI:** 10.3390/ijms23136997

**Published:** 2022-06-23

**Authors:** Bing Fu, Jinzeng Yang, Yan Yang, Jun Xia, Yinglin He, Qing Wang, Huihong Zhao, Huirong Yang

**Affiliations:** 1 College of Marine Sciences, South China Agricultural University, Guangzhou 510640, China; fub@stu.scau.edu.cn (B.F.); 20182067006@stu.scau.edu.cn (Y.Y.); 20202150006@stu.scau.edu.cn (Y.H.); wangqing@scau.edu.cn (Q.W.); zhaohh@scau.edu.cn (H.Z.); 2 Zhongshan Innovation Center of South China Agricultural University, Zhongshan 528400, China; 3 Department of Human Nutrition, Food and Animal Sciences, University of Hawaii at Manoa, Honolulu, HI 96822, USA; jinzeng@hawaii.edu; 4 Xinjiang Acadamy of Animal Sciences, Institute of Veterinary Medicine (Research Center of Animal Clinical), Urumqi 830000, China; xiajun2004263@163.com

**Keywords:** *Epinephelus coioides*, *myostatin*, nucleic acid vaccine, skeletal muscle

## Abstract

*Epinephelus coioides* is a fish species with high economic value due to its delicious meat, high protein content, and rich fatty acid nutrition. It has become a high-economic fish in southern parts of China and some other Southeast Asian countries. In this study, the *myostatin* nucleic acid vaccine was constructed and used to immunize *E. coioides*. The results from body length and weight measurements indicated the *myostatin* nucleic acid vaccine promoted *E. coioides* growth performance by increasing muscle fiber size. The results from RT-qPCR analysis showed that *myostatin* nucleic acid vaccine upregulated the expression of *myod*, *myog* and *p21* mRNA, downregulated the expression of *smad3* and *mrf4* mRNA. This preliminary study is the first report that explored the role of *myostatin* in *E. coioides* and showed positive effects of autologous nucleic acid vaccine on the muscle growth of *E. coioides*. Further experiments with increased numbers of animals and different doses are needed for its application to *E. coiodes* aquaculture production.

## 1. Introduction

*Myostatin*, also known as growth differentiation factor-8 (GDF-8), belongs to the transforming growth factor *β* superfamily. The human myostatin gene encodes 376 amino acids, including 3 exons and 2 introns. It is expressed in large amounts in skeletal muscle and its expression is also detected in other tissues and organs [1,2,3,4,5,6]. In addition, *myostatin* is present in mammals such as mice, rats, cattle, pigs, and goats [7,8,9,10,11]. The *myostatin* gene has been cloned and sequenced in a variety of fish including *Danio rerio* [12,13,14,15,16]. Past research indicates that myostatin is highly conserved among species, and its gene structure remains highly consistent across mammals [7]. Sequence alignment data show that the myostatin gene is conserved in the evolutionary processes. Myostatin in *Morone saxatilis*, *Oreochromis mossambicus*, *Sciaeops ocellatus*, mammals, and birds have the same amino acid ratio in the overall sequence from 60% to 65%; while the ratio in fish is the same. The ratios of amino acids are higher, and the similarities between striped seabass and tilapia are 93.4% and 96.8%, respectively. The conservation of this sequence implies that the fish myostatin gene may have a certain degree of similarity with mammalian species in function [17,18]. However, unlike mammals which only have one *myostatin* gene, bony fishes experience one more genome-wide duplication. Genome doubling has made some fishes have at least two *myostatin* genes [19]. In *D. rerio, myostatin-1* and *myostatin-2* exist in a variety of tissues, including skeletal and heart muscles, brain, liver, intestines, and ovary. The level of *myostatin-1* mRNA rises steadily throughout the development of the body, while the level of *myostatin-2* mRNA reaches its peak in the early stage of body development. The expression of *myostatin-1* is higher than *myostatin-2* in most tissues [20].

*Myostatin* negatively regulates muscle growth to a large degree [7]. “Double muscle phenotype,” caused by mutations of the *myostatin* gene, has been found in cattle, dogs, sheep, rabbits, and other mammals [8,21,22,23,24,25,26]. In *D. rerio*, knockout of the *myostatin gene* increased weight by 1.4 times in the experimental group after 4 months [27]. Researchers have a keen interest in regulating the muscle growth of related animals through the suppression of myostatin activity. Treatment with antibodies against myostatin resulted in increased skeletal muscle mass and grip strength in adult mice [28]. Skeletal muscle mass and endurance of mice can be enhanced through inoculation with a *myostatin* DNA vaccine (nucleic acid vaccine) [29]. Nucleic acid vaccines directly recombine the exogenous or autologous gene encoding an antigen protein with plasmid by DNA recombination technology, and then directly introduce the recombinant DNA into animal cells. This synthesizes the antigen protein through the transcription system of host cells to induce the host to produce an immune response to the antigen protein, to achieve the purpose of preventing and treating diseases [30]. Because fish autologous myostatin is weakly immunogenic, the antigen needs to be fused with the immune enhancement vector gene [31]. High immunogenicity fusion protein can be expressed by fusing genes such as follicular inhibin and somatostatin into immune enhancement vector genes such as HBsAg [32,33]. Therefore, based on our previous studies and experiences, we constructed a highly immunogenic myostatin autologous nucleic acid with HBsAg foreign gene. It is expected that this construct can stimulate the fish immune system to produce the corresponding antibody, which will inhibit the function of myostatin mature peptide, so as to promote muscle development and growth.

To understand the effects of the vaccine on muscle growth, we selectively studied myogenic regulatory proteins. Myogenic regulatory factors (*mrfs*) are a member of basic helix-loop-helix (bHLH) family of transcription factors [34], including myogenic factor 5 (*myf5*), myogenic differentiation (*myod*), myogenin (*myog*), and myogenic regulatory factor 4 (*mrf4*). These transcription factors control the myogenic commitment and differentiation of skeletal muscle cells during embryonic development and postpartum myogenesis. They act on multiple points in the muscle lineage by regulating proliferation, the irreversible cell cycle arrest of precursor cells, sarcomeres, and activation of muscle-specific genes to promote differentiation and sarcomere assembly, and jointly establishes skeletal muscle phenotype [35,36].

This study aims to develop a biological agent for making the *myostatin* nucleic acid vaccine to promote muscle growth and development, which will certainly decrease the cost of feeding, and improve the fillet meat yield. It can be developed as an alternative to transgenic animals. The effects of myostatin depression by its nucleic acid vaccine were studied on muscle growth of *E. coioides* and myofibers and related myogenic regulator factors. This is the first application of fish nucleic acid vaccine research to economically valuable aquatic species in the field.

## 2. Results

### 2.1. Construction of Nucleic Acid Vaccine

**Figure 1:**
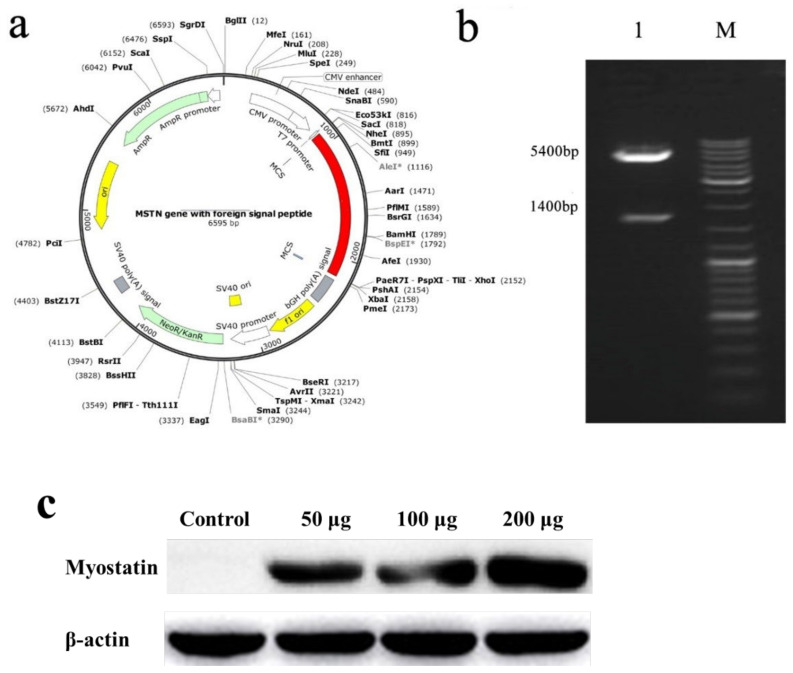
(**a**) Schematic diagram of nucleic acid vaccine. (**b**) Double enzyme digestion electropherogram. 1: Plasmid digestion band, 1400 and 5400 bp; M: DNA marker. (**c**) Western blotting analysis of HBSAg protein in muscle of vaccinated *E. coioides*.

The obtained exogenous gene (indicated by the red in Figure 1) was cloned into pcDNA3.1+ to obtain the recombinant plasmid, which was the nucleic acid vaccine (Figure 1a), and *Nhe I* and *Xho I* were used for double enzyme digestion identification. The final expectation was to obtain bands of 1400 and 5400 bp, which indicated that the plasmid was the target plasmid (Figure 1b). To confirm the expression of recombinant plasmid in muscle in vaccinated *E. coioides*, we performed western blot with anti-HBSAg antibody. As shown in Figure 1c. The result confirmed the expression of recombinant plasmid in muscle of vaccinated *E. coioides*. Furthermore, the HBSAg expressing in muscle increased along with the dose of recombinant plasmid injected, and the highest expression level of HBSAg was seen in the 200 μg vaccine group.

### 2.2. Effects of Myostatin Autologous Nucleic Acid Vaccine on Body Weight and Length

**Figure 2:**
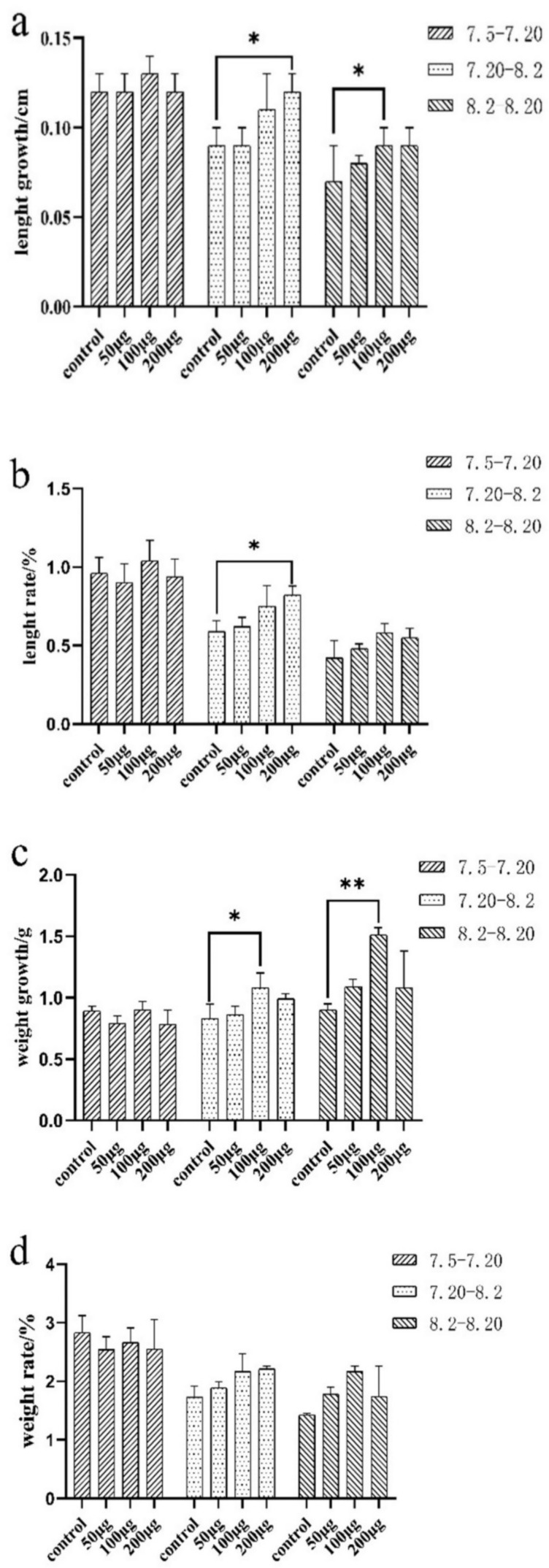
Growth and rate of length and weight of *E. coioides.* (**a**) Growth of the length of *E. coioides*; (**b**) rate of the length of *E. coioides*; (**c**) growth of the weight of *E. coioides*; (**d**) rate of the weight of *E. coioides*. Bar: mean ± SD. * *p* < 0.05 and ** *p* < 0.01 unpaired *t* test, n = 3.

The body weight and length of *E. coioides* at each immunization and final sampling were recorded and analyzed. The results showed that body weight and length of the three experimental groups of *E. coioides* did not change significantly from the first immunization to the second immunization (7.5–7.20) when compared with the control group. From the second to the third immunization (7.20–8.2) and the third immunization to the sampling period (8.2–8.20), the growth and rate of the body length and weight of the three experimental groups of *E. coioides* showed an upward trend with the increase in the injection dose in comparison with the control group. After statistical analysis, from the second injection to the third injection, the length growth and weight rate of the experimental group and the control group with a dose of 200 μg were significantly different. There was a significant difference in the weight growth between the experimental and control groups with an injection dose of 100 μg. From the third injection to the sampling period, the length and weight growth of the experimental group and the control group with an injection dose of 100 μg were significantly different (Figure 2).

### 2.3. Effects of Myostatin Autologous Nucleic Acid Vaccine on the Muscle Tissue

**Figure 3:**
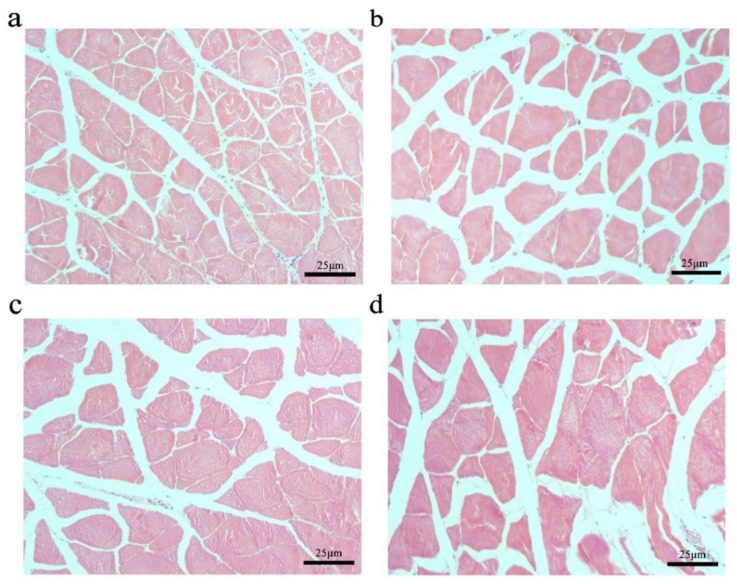
Changes in muscle fibers of *E. coioides* after immunization. (**a**) HE staining of paraffin sections of muscle fibers in the control group; (**b**) HE staining of paraffin sections of muscle fibers in the immunized 50 μg vaccine group; (**c**) HE staining of paraffin sections of muscle fibers in the immunized 100 μg vaccine group; (**d**) HE staining of paraffin sections of muscle fibers in the immunized 200 μg vaccine group.

**Figure 4:**
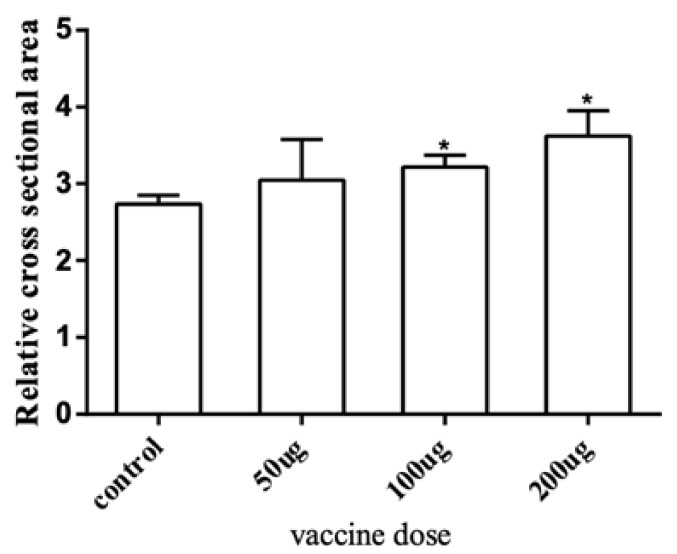
The average relative area of a single muscle fiber. The cross-section range of muscle fiber cells was accurately selected and the relative cross-sectional area of a single muscle fiber was calculated through Adobe Photoshop CS6 software. Bar: mean ± SE. * *p* < 0.05 unpaired *t* test, n = 4.

To further explore the effect of the *myostatin* autologous nucleic acid vaccine on the muscle tissue of *E. coioides*, the muscle fibers of *E. coioides* were made into paraffin sections, stained with H&E, and photographed under a microscope (Figure 3). Image processing was performed by Adobe Photoshop CS6 software, and average relative cross-sectional area of muscle fibers in the control group and the three experimental groups was calculated. It was found that the average relative cross-sectional area of muscle fiber in the experimental group was larger than that of the control group. With the increase in the immunization dose of the experimental group, the relative cross-sectional area of the average muscle fibers increased. Statistical analysis found that compared with the control group, the experimental group with an injection dose of 100 and 200 μg had significantly larger muscle fibers (Figure 4).

### 2.4. Effects of Myostatin Autologous Nucleic Acid Vaccine on the Downstream and Related Genes of the Myostatin Signaling Pathway

**Figure 5:**
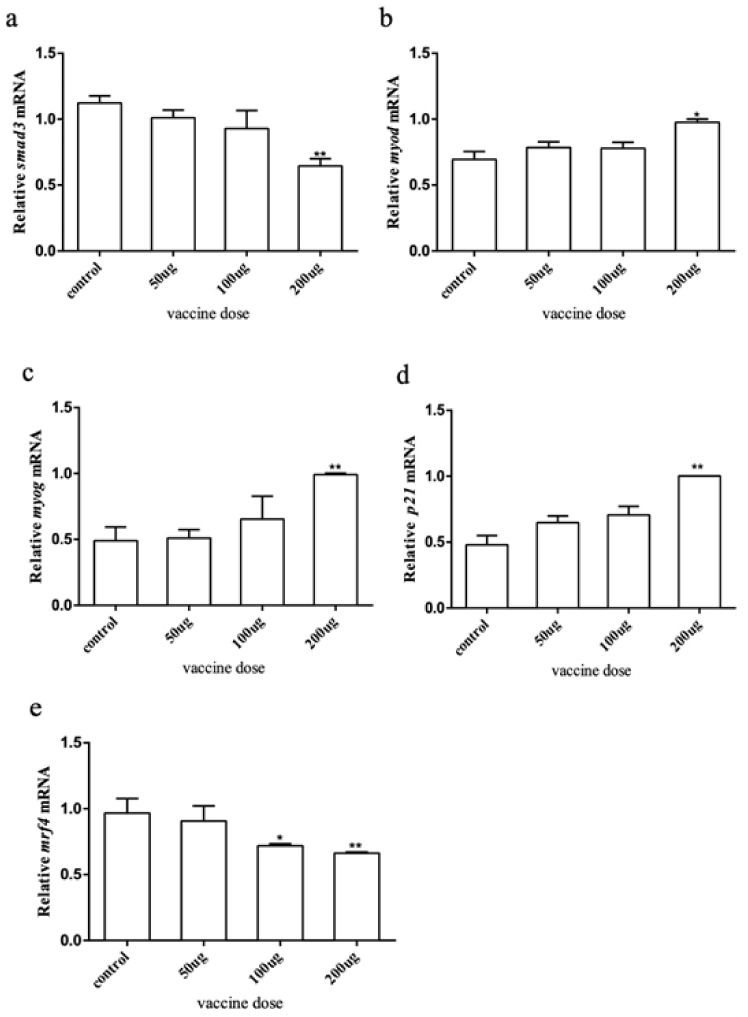
The expression of myostatin signaling pathway downstream and related genes by immune *myostatin* autologous nucleic acid vaccine. (**a**) Expression level of *smad3*; (**b**) expression level of *myod*; (**c**) expression level of *myog*; (**d**) expression level of *p21*; (**e**) expression level of *mrf4*. Bar: mean ± SE. * *p* < 0.05 and ** *p* < 0.01 unpaired *t* test, n = 4.

To explore how the myostatin autologous nucleic acid vaccine works on the muscles of *E. coioides*, muscle samples were taken from the experimental and control fish, and the total RNA was extracted and reverse transcribed into cDNA. RT-qPCR was used to detect the downstream genes of the *myostatin* signaling pathway and myogenic regulatory genes *smad3*, *myod*, *myog*, *p21,* and *mrf4*. The results showed that after *myostatin* autologous nucleic acid vaccine immunization, the mRNA expression of *smad3* and *mrf4* in the three doses of the experimental group showed a downward trend (Figure 5). After statistical analysis, there was no significant difference in *smad3* when the dose was 50 and 100 μg, and there was a significant difference when the dose was 200 μg. There was no significant difference in *mrf4* when the dose was 50 μg, and there was a significant difference when the dose was 100 and 200 μg. The mRNA expression levels of *myod*, *myog*, and *p21* all showed an upward trend, but there were significant differences between treatment and control groups when the dose was 200 μg.

## 3. Discussion

In this study, we explored the nucleic acid vaccine approach to depress the biological functions of myostatin in *E. coioides. E. coioides* was used as the experimental animal, and the immune experiment was carried out. Since there is currently no product on the market that can detect whether myostatin antibodies are produced in the body of *E. coioides*, the immune effects are analyzed in animal growth performances, myofiber and gene expression levels, and the antibodies were not analyzed. The growth rate of length and weight in one of the experimental groups had an upward trend compared with the control group. After statistical analysis, there were significant differences. After the immunization experiment, the experimental group and the control group were sampled, and muscle sections were made. Microscopic observation revealed that the average relative cross-sectional area of a single muscle fiber in the experimental group was larger than that of the control group, and there were significant differences. In previous studies, it had been demonstrated in a variety of animals that inhibiting the expression of *myostatin* could lead to an increase in the number of muscle fibers or hypertrophy and a significant increase in body weight [7,8,27,37,38]. Additionally, a study using zebrafish as a model organism for myostatin knockout showed that after interference with *myostatin*, the weight and body length growth rate of the experimental group showed an upward trend and the phenomenon of thick muscle fibers appeared. This is the same as most other research results. Therefore, it could be determined that the *myostatin* autologous nucleic acid vaccine promotes muscle growth of *E. coioides*. However, in-depth analysis of the growth data of the body length and weight of this research object revealed that there were only some significant differences. There were several speculations about this: first, referring to the period during the first injection to the second injection in this experiment, the growth rate of body weight and length of the three experimental groups did not change significantly compared with the control group. With the extension of time, after repeated immunizations during the subsequent farming period, the rate began to show an upward trend. Therefore, in future studies, we will consider extending the breeding time and increasing the immunization times to better promote muscle growth of *E. coioides*. Secondly, most of the past studies focused on mammals, and the types and numbers of fish were relatively few. However, this study is the first to use the *E. coioides* as the research object and it was speculated that the difference in the species causes the function of myostatin to be not completely the same. Studies have shown that in some animals *myostatin* was specifically expressed only in muscle tissue [7], while weak expression of *myostatin* was detected in some tissues such as the heart, spleen, kidney, and breast, the expression of direct homolog of *myostatin* was detected in almost all tissues of fish [39,40,41]. The different expression patterns of *myostatin* in fish and mammals means that its function may also be different. Fish *myostatin*-related articles also point out that there was no clear correlation between fish muscle growth rate and the expected *myostatin* expression level, which makes us doubt its role in the regulation of fish muscle growth. Fish *myostatin* could be used as a general inhibitor of cell proliferation and cell growth to control tissue quality, but it could not be used as a strong muscle regulator [17].

The muscle samples were taken from all groups, and the RNA was extracted and reverse transcribed into cDNA to detect the expression of the *myostatin* signaling pathway downstream and related genes. As mentioned above, *myostatin* was mediated by Smads [42,43,44,45,46], and then regulated the expression of *mrfs*. The expression of Smad3 decreased, the expression of *myog* and *myod* were upregulated, and the expression of *mrf4* decreased after immunization with *myostatin* autologous nucleic acid. It was speculated that *myostatin* autologous nucleic acid vaccine can produce corresponding protein antibody in *E. coioides*, neutralizing endogenous protein and reducing the expression of Smad3 in the pathway; thus, upregulating the downstream *myod* and *myog*, which was consistent with the experimental results of sheep and mice immunized with *myostatin* autologous nucleic acid vaccine [47]. In this study, the body length/body weight growth speed, length/weight growth rate and cross-sectional area of muscle fiber increased after immunization with *myostatin* nucleic acid vaccine, which indicated that *myostatin* nucleic acid vaccine played a certain role in the growth of *E. coioides* in this study. On the other hand, the expression of *p21* in this experiment showed an upward trend, while that of *mrf4* showed a downward trend, which was different from the results of other studies that inhibited the function of *myostatin*. According to the existing literature, we speculated that there were several possibilities. First, in this study, pcDNA 3.1+ vector was used to construct *myostatin* nucleic acid vaccine and to inject it into *E. coioides*, while previous studies mostly used siRNA or lentiviral vector to treat cells. Secondly, the primary muscle cells of *E. coioides* were used in this study, but previous studies were mostly found in goats, mice, pigs, and other species. Finally, it may be that differentiation of experimental cells was triggered in the late growth stage, leading to the upregulation of *p21* expression. When the myoblasts gradually fused to form myotubes, and the myotubes gradually differentiated into myofibers, *mrf4* expression tended to be stable and gradually decreased. The higher the maturity and integrality of muscle fibers, the lower the expression level of *mrf4* [47,48]. In another study we are preparing to publish, we found that Myostatin recombinant protein regulates cell differentiation by regulating the expression of *smad3*, *mrf4*, *myod,* and *myog*; at the same time, Myostatin recombinant protein regulates the expression of *p21* to inhibit cell proliferation. The biological function of Myostatin at the level of muscle cells was verified.

In this study, the body weight and length of *E. coioides* increased to a certain extent after immunization with nucleic acid vaccine, and the cross-sectional area of muscle fiber also increased significantly. At the same time, the expression of related genes downstream of the *myostatin* signaling pathway changed in previous studies. This shows that the *myostatin* nucleic acid vaccine could promote the muscle growth of *E. coioides*. However, considering that the increase in body weight and body length was not as significant as that of mammals, and the sampling location of muscle slices is the immune site, it was impossible to determine whether the increase in cross-sectional area of muscle fibers has an overall effect. Additionally, the change in body weight and body length might be caused by the change in immune site. Therefore, it was not confirmed that this promotion effect was very strong at present, which needs further exploration.

## 4. Materials and Methods

### 4.1. Construction of Nucleic Acid Vaccine

The DNA construct (Figure 1a) consists of KOZAK sequences, signal peptide of Japanese encephalitis virus, Surface core antigen of hepatitis B virus, Th epitope, somatostatin, linker, and *myostatin* mature peptide, which was designed according to the vector specification and existing research results. KOZAK sequence increases the expression of foreign protein; signal peptide of Japanese encephalitis virus correctly guides foreign proteins and improves protein biological efficiency; Th epitope improves protein immunogenicity; Surface core antigen of hepatitis B virus makes the myostatin mature peptide exogenous; Somatostatin and *myostatin* are genes that inhibit muscle growth; linker sequence helps hepatitis B surface antigen protein and myostatin mature peptide protein appropriately linked together. After being synthesized by Sangon Biotech, it was cloned into pcDNA3.1+ vector, and finally, the recombinant plasmid and positive bacteria were obtained. The recombinant plasmids were identified by double digestion with *Nhe I* and *Xho I*. See Table 1 for gene sequence.

### 4.2. Vaccine Production

The positive bacteria obtained above were inoculated into a large amount of LB medium for expanded culture and plasmid was extracted by alkali lysis. After obtaining a large number of crude extract particles, the plasmid was purified by tangential flow of hollow fiber filtration system Akta flux, CFP-4-E-2U hollow fiber through the filter column, tangential flow of hollow fiber filtration system Akta flux, and UFP-300-C-2U hollow fiber through the filter column. The specific steps are described above [49].

### 4.3. Animals

*E. coioides* were collected from the Guangdong Marine Fisheries Test Center (22°705093′ N, 114°541433′ E) China, on 4–6 March 2020. Weight 31.6 ± 6.7 g, length 12.8 ± 1.0 cm. Before the experiment, the fish were cultured in a mariculture system at 25 °C for two weeks. After the culture, the obtained samples were placed in the centrifuge tubes with Sample Protector for RNA/DNA (TAKARA, Kyoto, Japan), and then stored at −80 °C. The other part of the sample was placed in Bouin’s Fixative Solution (PHYGENE, Fuzhou, China). Voucher specimens were deposited at the Department of Aquatic Animal Medicine, College of Marine Sciences, South China Agricultural University, Guangzhou, China (Accession number 20200824). All animal experiments were conducted by the guidelines and approval of the Animal Research and Ethics Committees of South China Agricultural University (#2019-0136).

### 4.4. Immunization Protocol and Sample Collection

A total of 180 *E. coioides* about 13 cm in length were randomly divided into four groups, with three parallel groups in each group. One group was injected with 100 μL of saline as the control group, and the other three groups were injected with 100 μL of *myostatin* autologous nucleic acid vaccine at concentrations of 500, 1000, and 2000 ng/μL as the experimental group. All *E. coioides* were fed under the same conditions and ate freely. The first immunization was completed three times, and each group was immunized three times with an interval of 2 weeks. After the third immunization, the animals were cultured for 2–4 weeks, fasting for one day before sampling. When sampled, the weight and length of all subjects were recorded. Then, the muscle tissue was taken and one part stored in Bouin’s Fixative Solution (PHYGENE, Fuzhou, China) and the other part in Sample Protector for RNA/DNA (TAKARA, Kyoto, Japan).

### 4.5. Muscle Histology

The muscles were fixed overnight in Bouin’s Fixative Solution (PHYGENE, Fuzhou, China) at room temperature, and then serially sectioned after dehydration in an ethanol gradient, transparent in xylene, and embedded in paraffin. The slice thickness was 5 μm. The chips were then dipped in two cylinders of xylene for 2 min, 10 times each time. After de-waxing, gradient concentration ethanol rehydration was carried out, and H&E staining was carried out after rehydration. The slides were dehydrated and dried, and finally sealed with neutral gum. Slides were observed and photographed under a Motic optical microscope.

### 4.6. RNA Extraction, cDNA Synthesis and qRT-PCR

Total RNA from tissue was extracted by using Trizol reagent (Invitrogen, CA, California, USA). Reverse transcription reactions were performed using a ReverTra Ace^®^ qPCR RT Kit (TOYOBO, Osaka, Japan) using the protocol provided by the manufacturer. RT-qPCR was performed using a SYBR^®^ Green Realtime PCR Master Mix (TOYOBO, Osaka, Japan) using the protocol provided by the manufacturer. The reference genes *β-actin* were used, and qRT-PCR gene targets and primers are given in Table 2. 

### 4.7. Western Blot Analysis

To demonstrate that the recombinant plasmid was really expressed in muscle cells of injected fish, we perform a western blot with anti-HBSAg antibody (10-1323, Fitzgerald Industries International, North Acton, MA, USA).The mixed muscle tissues from three fish of each group were used for Western blot analysis. Protein was extracted from muscle tissue and the protein concentration was evaluated using a BCA protein assay. Equal amounts of protein were subjected to SDS-PAGE and transferred onto polyvinylidene difluoride (PVDF) membranes (Millipore, Burlington, MA, USA). Immunoblotting was carried out using a 1:1000 dilution of antibodies anti-HBSAg (Abcam, Cambridge, UK) and anti-β-actin (Abcam) according to the manufacturer’s instructions. Primary antibodies were visualized using the enhanced chemiluminescent development reagent (Amersham Pharmacia Biotech Ltd., Little Chalfont, UK) following the peroxidase-conjugated secondary antibody (Santa Cruz Biotechnology, Inc., Dallas, TX, USA).

### 4.8. Statistical Analysis

The experimental data was statistically analyzed using Graphpad Prism v8.0.2.263 (GraphPad Software, San Diego, CA, USA), with *β*-actin as an internal parameter and analyzed by 2^−^^ΔΔ^^Ct^ method. Data are expressed as mean ± standard error (Mean ± SE) or mean ± standard deviation (Mean ± SD). Statistical significance was defined as *p* < 0.05. The cross-sectional image of muscle fiber cells was used to accurately select the cross-section range of muscle fiber cells and calculate the relative cross-sectional area of a single muscle fiber through Adobe Photoshop CS6 software. The calculation formula is as follows. Then, statistical analysis was performed by Graphpad Prism 8.0 software.

$$S=\frac{\mathrm{Pixels}\;\times \;\mathrm{Width}\;\left(\mathrm{Document}\;\mathrm{Size}\right)\times \mathrm{Height}\;\left(\mathrm{Document}\;\mathrm{Size}\right)}{n\;\times \;\mathrm{Width}\;\left(\mathrm{Pixel}\;\mathrm{Dimensions}\right)\times \;\mathrm{Height}\;\left(\mathrm{Pixel}\;\mathrm{Dimensions}\right)}$$

## Figures and Tables

**Table 1:** Gene sequence used to construct nucleic acid vaccine.

Name	Sequence (5′–3′)
KOZAK	GCCACC
signal peptide of Japanese encephalitis virus	ATGGGAAAGAGAAGCGCCGGCAGCATCATGTGGCTGGCCAGCCTGGCCGTCGTGATCGCCTGTGCCGGCGCC
Surface core antigen of hepatitis B virus	ATGGAGAACATCGCATCTGGACTCCTTGGACCCCTGCTCGTGCTGCAGGCCGGGTTTTTCCTGCTGACAAGAATCCTCACAATACCACAGAGTCTCGACTCCTGGTGGACTTCTCTCAATTTTCTCGGGGGAACACCCGTGTGCCTTGGCCAAAACTCCCAGTCCCAAACCTCCAGTCATAGCCCAACCTGCTGTCCTCCAATTTGTCCTGGTTATCGCTGGATGTGTCTGCGGAGGTTTATCATCTTTCTCTGCATCCTGCTGCTCTGCCTCATCTTCCTGCTGGTTCTTCTGGACTATCAGGGTATGCTGCCCGTTTGTCCTCTCATTCCAGGAAGCAGCACGACCAGCACCGGCCCATGCAAGACCTGCACCAGTCCTGCTCAGGGCACCTCTATGTTTCCCAGCTGCTGTTGTACTAAACCTACGGATGGCAACTGCACCTGCATTCCCATCCCAAGCTCTTGGGCTTTCGCCAAGTACCTCTGGGAATGGGCCAGCGTCCGATTCTCTTGGCTCAGTCTGCTCGTGCCTTTTGTGCAGTCCTTCGTGGGTCTTTCCCCCACTGTCTGGCTTAGTGTGATATGGATGATGTGGTACTGGGGCCCTAGTCTGTACAACATCCTGAGTCCCTTCATACCGCTGCTGCCGATATTCTTTTGCCTTTGGGTCTAC
Th epitope	CAGTATATAAAAGCAAATTCTAAATTTATAGGTATAACTGAA
somatostatin	GCCGGCTGTAAGAACTTCTTTTGGAAAACATTCACCAGCTGC
linker	GGAGGCGGTGGATCCGGAGGCGGAGGAAGCGGAGGTGGCGGCTCT
myostatin mature peptide gene	TCAGGCCTGGACTGTGACGAGAACTCTCCAGAGTCCCGGTGCTGCCGCTATCCGCTCACAGTGGACTTTGAAGACTTTGGCTGGGACTGGATTATTGCCCCAAAGCGCTACAAGGCCAACTATTGCTCCGGGGAGTGTGAGTACATGCACTTGCAGAAGTACCCACACACCCACCTGGTGAACAAAGCCAATCCCAGAGGGACCGCAGGCCCCTGCTGCACCCCCACCAAGATGTCACCCATCAACATGCTCTACTTTAACCGAAAAGAGCAGATCATCTATGGCAAGATCCCCTCCATGGTGGTGGACCGTTGTGGATGCTCTTGA

**Table 2:** Primers used in this study.

Primer	Sequence (5′-3′)
*myod*-RT-F	CGGCGGCTTGGTAA
*myod*-RT-R	CATCGGAGCAGTTGGA
*p21*-RT-F	GGTGGTAGAAAGAAAGAT
*p21*-RT-R	TTGCCTGTAGAGTCGTA
*β*-actin-RT-F	TCTTCCAGCCATCCTTCCTTGG
*β*-actin-RT-R	CTGCATACGGTCAGCAATGCC
*myog*-RT-F	GATGGGCTTATGTGGG
*myog*-RT-R	GGTAACCGTCTTCCTTTT
*mrf4*-RT-F	TACAACGGCAACGACA
*mrf4*-RT-R	GCCCACATGAGGCACT
*smad3*-RT-F	GCATAACCATACCCAGAT
*smad3*-RT-R	CGCAGACTTCGTCCTT

## Data Availability

All data discussed are contained in the manuscript.
